# Nationwide Patterns and Predictors of Sick Leave Among Healthcare Workers in Kuwait, 2022

**DOI:** 10.3390/healthcare14060758

**Published:** 2026-03-18

**Authors:** Saleh Alsarhan, Lolwah Alzafiri, Eiman Alawadhi

**Affiliations:** 1 Department of Health Policy and Management, College of Public Health, Kuwait University, Kuwait City 13060, Kuwait; lolwah.alzafiri@ku.edu.kw; 2 Kuwait Center for Disease Control and Prevention (KCDC), Ministry of Health, Kuwait, Kuwait City 13001, Kuwait; 3 Department of Epidemiology and Biostatistics, College of Public Health, Kuwait University, Kuwait City 13060, Kuwait; eiman.alawadhi@ku.edu.kw

**Keywords:** health personnel, sick leave, absenteeism, influenza vaccines, occupational health

## Abstract

**Background/Objectives**: Healthcare worker (HCW) sickness absenteeism can disrupt healthcare service delivery and increase workload pressures, yet evidence from Kuwait remains limited. This study examined patterns of sick leave episodes and duration among HCWs in Kuwait and identified associated predictors. **Methods**: We conducted a nationwide retrospective analysis of sick leave utilization using Ministry of Health (MOH) administrative records for 2022, including 51,204 HCWs across all MOH healthcare facilities. Outcomes were sick leave episodes and sick leave duration. Independent variables included age, gender, nationality, place of residence, profession, managerial position, and influenza vaccination status. Zero-inflated negative binomial regression models were used to estimate adjusted incidence rate ratios (IRRs) with 95% confidence intervals (CIs). **Results**: In 2022, 196,840 sick leave episodes and 295,206 sick leave days were recorded, with 53% of HCWs experiencing at least one episode. Upper respiratory tract infection (URTI)-related sick leave exhibited seasonal variation, with higher proportions during winter months. Younger age, female sex, Kuwaiti nationality, non-managerial position, and medical technician professions were associated with higher sick leave episodes and duration, while physicians, dentists, and pharmacists had lower sick leave utilization compared with nurses. Influenza vaccination was associated with fewer sick leave episodes and shorter duration. **Conclusions**: Sick leave patterns among HCWs in Kuwait show noticeable seasonal, demographic, and occupational variation. Targeted preventive strategies and workforce policies may help reduce sick leave burden.

## 1. Introduction

Absenteeism among healthcare workers (HCWs) is a significant occupational and public health concern, particularly in health systems that rely on continuous and consistent clinical staffing. Sick leave or sickness absence can be defined as “absence from work that is attributed to sickness by the employee and accepted as such by the employer” [1]. Although sick leave is intended to safeguard both employees and patients, it can disrupt workflow, lengthen patient wait times, and place substantial strain on healthcare staff and service delivery [2]. High levels of absenteeism may also increase operational costs and reduce workforce productivity within healthcare systems. Among HCWs, the risk of sick leave has been consistently greater than that of employees in many other industries, largely due to increased exposure to infectious diseases, demanding work situations, and shift patterns [2,3,4].

During the COVID-19 pandemic, systemic weaknesses in labor capacity were highlighted, raising awareness of sick leave in healthcare settings. International studies revealed that significant increases in sick leave due to respiratory illnesses were documented during periods of peak transmission, with higher absenteeism extending past the initial phase of the pandemic [5,6]. According to Elabd et al. (2020) and Popa et al. (2025), respiratory infections, particularly viral upper respiratory tract infections (URTIs), remain among the main occupational causes of sick leave among HCWs, even after the pandemic subsided [3,7]. These infections also contribute to seasonal fluctuations in workforce availability and frequent short-term absences.

Previous occupational health research has indicated that sick leave is not evenly distributed within the healthcare workforce. Higher rates of sick leave have been consistently reported among younger workers, females, frontline clinical staff, and non-managerial and lower-grade HCWs [4,8]. Preventive measures, such as seasonal influenza vaccination, have also been linked to absenteeism and shorter sick leave duration among HCWs [3,9].

In Kuwait, the healthcare system is predominantly government-funded, operated, and financed by the Ministry of Health (MOH), which employs the majority of HCWs and delivers publicly funded healthcare services [10]. While direct nationwide evidence from Kuwait is limited, studies from neighboring Gulf and Middle Eastern settings consistently indicate that HCW absenteeism varies by profession and occupational role, and that respiratory infections represent a leading cause of sick leave, particularly during the COVID-19 period [3,4,11]. However, most available studies in the region have been limited to single institutions or specific occupational groups, restricting their ability to provide system-wide insights into healthcare workforce absenteeism. To date, no national-level study has evaluated sick leave patterns and their demographic and occupational correlates across Kuwait’s healthcare workforce. This gap hinders the ability to identify high-risk occupational groups and guide evidence-based workforce planning as well as targeted risk-reduction initiatives within occupational health systems. Therefore, this study aimed to examine national patterns of sick leave among HCWs employed by the MOH in Kuwait in 2022 and to identify demographic and occupational predictors of both sick leave episodes and sick leave duration.

## 2. Materials and Methods

A nationwide retrospective observational study of sick leave utilization among HCWs was conducted using MOH administrative records for the year 2022, covering all MOH healthcare facilities in Kuwait. The year 2022 was selected as it represents the most recent complete dataset prior to major policy and system changes in Kuwait, including the introduction of online self-certified sick leave in 2023 and the planned implementation of smart fingerprint attendance systems [12,13]. Sick leave records in the administrative dataset originate from physician-certified sick leave certificates issued during clinical visits at MOH facilities. Diagnoses and duration of sick leave are documented by the attending physician at the time of consultation and subsequently entered into the MOH administrative information system by authorized clinical staff. The datasets included HCWs’ demographic characteristics (age, gender, nationality, and place of residence), occupational characteristics (profession and managerial position), sick leave records (date of sick leave episode, duration of sick leave in days, and recorded diagnoses), and 2021–2022 influenza vaccination status. The initial dataset contained records for 51,459 HCWs. After exclusion of records with missing or inconsistent information in key demographic or sick leave variables (255 records; 0.5% of the initial dataset), a total of 51,204 HCWs were used for the final analysis representing all major healthcare professions within the MOH workforce. The workforce counts in the dataset were broadly consistent with the MOH Annual Health Report 2022 [14]. The resulting analytic dataset contained no missing data for variables included in the descriptive and regression analyses. All records were fully de-identified prior to analysis.

The two primary outcomes of this study were sick leave episode and sick leave duration. Sick leave episode (captures frequency) was defined as the number of distinct sick leave episodes recorded for each HCW during 2022, irrespective of episode duration or diagnosis. Contiguous sick leave days covered under a single certificate were treated as one episode, with duration defined as the total number of certified days. Sick leave episodes that initiated in 2021 and continued into 2022 (*n* = 6 episodes) were included because part of their certified duration occurred within the 2022 study period. Sick leave duration was defined as the cumulative number of sick leave days recorded for each HCW during the year 2022. In addition, we measured the prevalence of sickness absence which was calculated as the proportion of HCWs who experienced at least one sick leave episode during the study year. Sick leave episodes were further classified as URTI-related or non-URTI-related based on diagnostic descriptions often recorded as free-text entries in the administrative dataset. Prior to classification, all diagnostic text fields were standardized through a cleaning process that included converting entries to lower case, removal of punctuation and extra spaces, and correction of common spelling variations. Episodes were then screened using a predefined list of keywords representing upper respiratory tract infections, including terms such as “URTI,” “upper respiratory infection,” “common cold,” “influenza,” “flu,” “pharyngitis,” “tonsillitis,” and “laryngitis.” Records containing any of these terms were classified as URTI-related sick leave episodes.

Independent demographic variables included age, which was measured as of 1 January 2022, and was analyzed both as a continuous variable and as a categorical variable (≤29, 30–39, 40–49, and ≥50 years) for descriptive and comparative analyses. The demographic variables also included gender (male, female), nationality (Kuwaiti, non-Kuwaiti), and place of residence (Asimah, Hawalli, Farwaniya, Mubarak Al–Kabeer, Ahmadi, and Jahra). Independent occupational variables included managerial position (managerial vs. non-managerial) and profession (nurses, physicians, dentists, pharmacists, medical technicians, others). Managerial position was included because organizational hierarchy and supervisory responsibilities have been shown in previous occupational health studies to influence sickness absence patterns and workplace flexibility [4]. Medical technicians included those working in laboratory, radiology, nutrition and feeding, physiotherapy, pharmacy, emergency medical, environmental, anesthesia, sterilization, dental, speech and hearing, occupational and respiratory therapy, psychotherapy, and other allied health technical specialties. The “other” category included non-technical, vocational, and service personnel (e.g., clerical staff, maintenance workers, and ancillary healthcare support roles). The profession variable was categorized based on the MOH workforce classification system [14]. Influenza vaccination status was also included as an independent variable and defined as receipt of the influenza vaccine, as documented in administrative records, at any time between October 2021 (start of the 2021–2022 MOH winter vaccination campaign) and December 2022 (end of the study period). Vaccination status was examined as a potential occupational health factor influencing sickness absence among HCWs.

The main data analytical approaches used for this study were descriptive tabulations and multivariable regression analyses. Descriptive statistics were used to summarize HCW characteristics and sick leave outcomes. Age was analyzed both as a continuous variable (mean and standard deviation (SD)) and as categorical age groups (frequencies and percentages). Sick leave episodes and duration (days) were reported using medians and interquartile ranges (IQRs) because both variables showed highly right-skewed distributions with a large proportion of zero values. Categorical variables were summarized using frequencies and percentages. Also, monthly sick leave episodes were summarized descriptively and plotted to examine temporal patterns throughout 2022. Furthermore, the proportion of HCWs with and without sick leave episodes was calculated for each profession and for the overall study population.

Zero-inflated negative binomial (ZINB) regression models were used because the sick leave outcomes were count variables characterized by overdispersion and a substantial proportion of zero observations (HCWs with no recorded sick leave during the study year). Inspection of the outcome distributions showed a high proportion of zero counts and substantial overdispersion (variance exceeding the mean), supporting the use of a ZINB model. Model comparison using the Akaike Information Criterion (AIC) and Bayesian Information Criterion (BIC) demonstrated improved fit for the ZINB specification compared with standard negative binomial models (Table S1 in the Supplementary Materials). The ZINB model includes two components: a count component estimating the expected number of sick leave episodes or days, and a logit inflation component estimating the probability of belonging to an excess-zero group. Both components included the same demographic and occupational predictors (age, gender, nationality, place of residence, profession, managerial position, and influenza vaccination status). Results from the count component are presented as adjusted incidence rate ratios (IRRs) with 95% confidence intervals, while the inflation component results are provided in the Supplementary Materials (Table S2). Adjusted predicted values and corresponding 95% CIs were estimated from the count component of the ZINB models to illustrate expected sick leave episodes and days across key characteristics. Predictions were generated using marginal standardization, whereby the variable of interest (e.g., age, gender, profession, or residence) was set to specific values while all other covariates were retained at their observed values and averaged over the study population. These estimates represent the model-predicted mean number of sick leave episodes or days for HCWs with the specified characteristic. Because the outcomes are highly right-skewed, the model-based predicted values represent adjusted means rather than medians and may, therefore, appear higher than the descriptive median values. Statistical significance was defined as *p* < 0.05. All analyses were conducted using Stata version 14.2 (StataCorp, College Station, TX, USA).

## 3. Results

### 3.1. Study Population and Characteristics

A total of 51,204 HCWs were included in the analysis, accounting for 196,840 sick leave episodes and 295,206 sick leave days in 2022. This corresponds to an average of 5.77 sick leave days per HCW per year (295,206 total days ÷ 51,204 HCWs). The median of sick leave episodes was 1 [IQR 5] and the median duration was 1 day [IQR 8]. The mean (SD) age was 36.21 (10.06) years, with the largest proportion (40.22%) aged 30–39 years. Females accounted for 61.87% of the HCWs, and 70.66% of the study population were non-Kuwaiti nationals. The highest proportion of HCWs resided in Farwaniya Governorate (30.15%), followed by Hawalli (26.84%), while Mubarak Al–Kabeer had the lowest proportion (4.36%). Nurses represented the largest profession (40.06%), followed by medical technicians (23.09%) and physicians (22.49%). Managerial positions were held by 1.20% of HCWs, while 7.31% of the study population received influenza vaccination (Table 1).

### 3.2. Sick Leave Burden and Distribution

**Figure 1:**
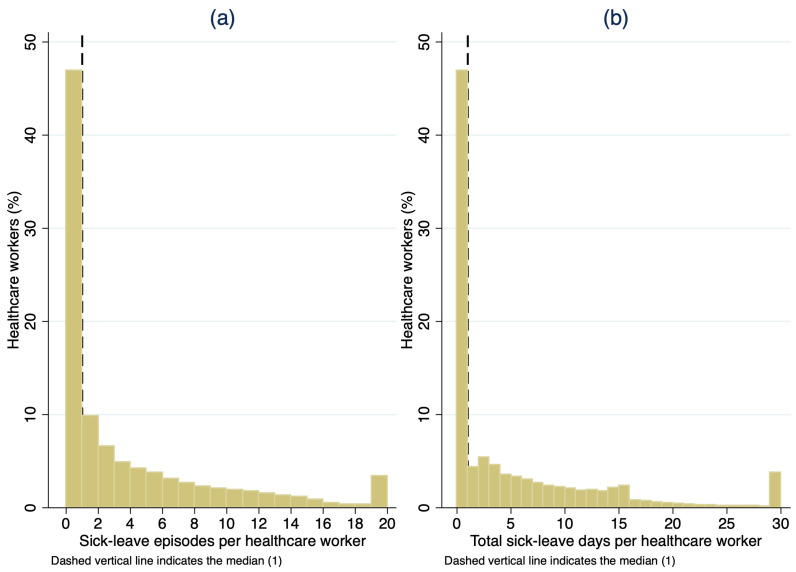
Distribution of sick leave episodes and total sick leave days among healthcare workers in Kuwait, 2022: (**a**) number of sick leave episodes per healthcare worker; (**b**) total sick leave days per healthcare worker. Note: Both outcomes demonstrate a highly right-skewed distribution with a large proportion of healthcare workers recording no sick leave episodes. For visualization purposes, extreme values were capped at 20 episodes and 30 days; however, all statistical analyses were conducted using the full uncapped dataset.

Figure 1 presents the distributions of both sick leave episodes and sick leave duration in days among HCWs in 2022. Both outcomes were highly right-skewed, with a large portion of HCWs recording no sick leave during the study period, indicating the presence of excess zeros and overdispersion.

### 3.3. Variation in Sick Leave Patterns

**Figure 2:**
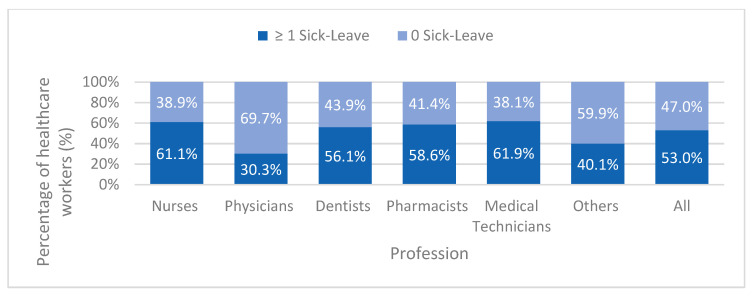
Distribution of healthcare workers with and without sick leave episodes by profession, Kuwait, 2022.

**Figure 3:**
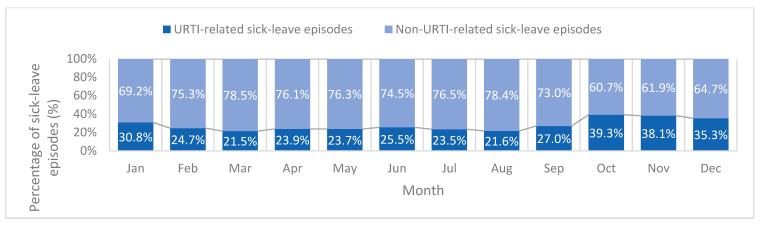
Monthly proportion of URTI-related and non-URTI-related sick leave episodes among healthcare workers in Kuwait, 2022 (N = 196,840 episodes). Percentages represent the proportion of URTI and non-URTI-related episodes relative to the total number of sick leave episodes recorded in each month.

Figure 2 shows the distribution of HCWs with and without sick leave episodes by profession. Overall, 53% of HCWs had at least one sick leave episode in 2022, while 47% had none. Among HCWs with at least one recorded sick leave episode, the average number of sick leave days was 10.9 per year. The proportion of workers with ≥1 sick leave episode was highest among medical technicians (61.9%) and nurses (61.1%), followed by pharmacists (58.6%) and dentists (56.1%). In contrast physicians had the lowest proportion of sick leave utilization, with only 30.3% reporting at least one sick leave episode. Furthermore, the distribution of sick leave episodes varied across the calendar year, with URTI-related episodes exhibiting clear seasonal pattern, with higher proportions in the winter months (Figure 3). The proportion of URTI-related sick leave rose sharply in October (39.3%) and November (38.1%) and remained elevated in December (35.3%) (Figure 3).

### 3.4. Predictors of Sick Leave Episodes and Duration (Multivariable Analysis)

The multivariable ZINB models identified several demographic and occupational predictors of sick leave episodes and duration (Table 2). Age was inversely associated with both outcomes, with each additional year of age associated with a 3.2% decrease in sick leave episodes and a 0.6% decrease in sick leave duration. Female HCWs had 22% more sick leave episodes and 11% longer sick leave duration than male HCWs after adjustment for other variables. Non-Kuwaiti HCWs had substantially fewer sick leave episodes and shorter sick leave duration than Kuwaiti HCWs. Differences were also observed across governorates and professions, with physicians, dentists, and pharmacists demonstrating lower sick leave utilization than nurses, while medical technicians showed higher levels. In addition, HCWs in managerial positions and those who received influenza vaccination had fewer sick leave episodes and shorter sick leave duration. Full model estimates are presented in Table 2 and the ZINB regression outputs of the inflation components are provided in the Supplementary Materials (Supplemental Table S2).

### 3.5. Adjusted Predicted Sick Leave Patterns

**Figure 4:**
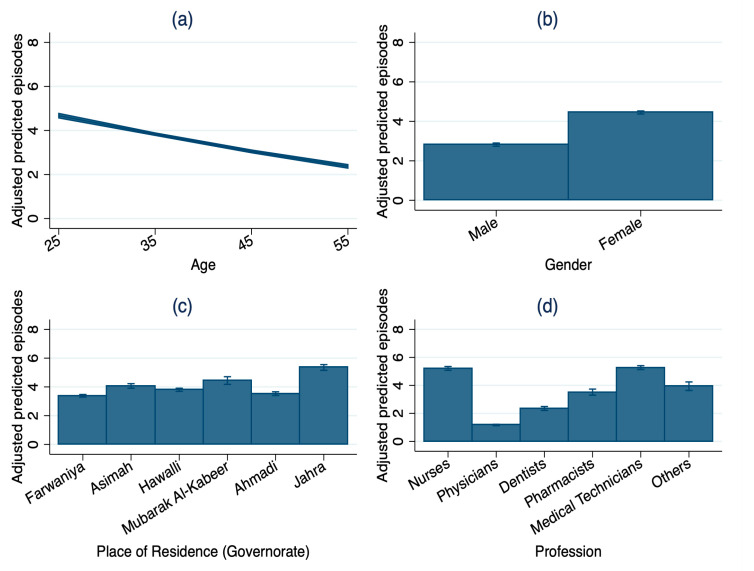
Adjusted predicted sick leave episodes by key characteristics of healthcare workers in Kuwait, 2022 (N = 51,204): (**a**) age; (**b**) gender; (**c**) place of residence (governorate); (**d**) profession. Note: Adjusted predicted values with 95% confidence intervals derived from zero-inflated negative binomial regression model. Predicted values represent model-based mean estimates and, therefore, may differ from the descriptive medians reported in Table 1.

**Figure 5:**
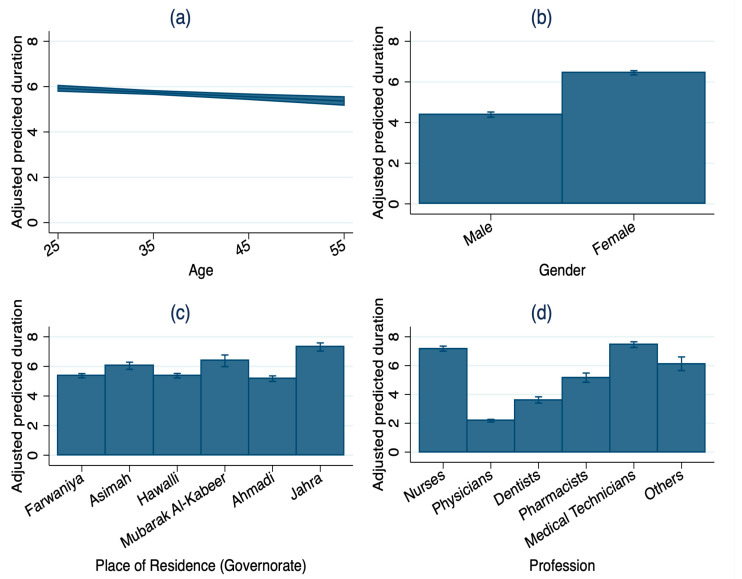
Adjusted predicted sick leave duration by key characteristics of healthcare workers in Kuwait, 2022 (N = 51,204): (**a**) age; (**b**) gender; (**c**) place of residence (governorate); (**d**) profession. Note: Adjusted predicted values with 95% confidence intervals derived from zero-inflated negative binomial regression model. Predicted values represent model-based mean estimates and, therefore, may differ from the descriptive medians reported in Table 1.

The adjusted predicted values represent model-based mean estimates derived from the ZINB regression models and, therefore, differ from the descriptive medians reported in Table 1. After adjustment of all covariates, younger HCWs had higher predicted sick leaves than older workers; a 25-year-old worker was predicted to have 4.67 sick leave episodes and 5.93 sick leave days per year, compared with 2.37 sick leave episodes and 5.36 sick leave days for a 55-year-old HCW (Figure 4 and Figure 5). Female HCWs were predicted to have 4.45 episodes and 6.45 days per year, while male HCWs were predicted to take 2.82 episodes and 4.39 days per year. Among professions, medical technicians and nurses had the highest predicted number of sick leave episodes and the longest duration (medical technicians 5.27 episodes; 7.45 days; nurses 5.21 episodes; 7.18 days), whereas physicians had the lowest (1.17 episodes; 2.18 days). Across governorates, workers residing in Jahra had the highest predicted number of sick leave episodes and the longest duration (5.44 episodes; 7.30 days), whereas Farwaniya had the lowest episodes, and Ahmadi had the lowest duration (Farwaniya: 3.38 episodes, 5.37 days; Ahmadi: 3.53 episodes, 5.17 days).

## 4. Discussion

This nationwide study examined patterns of sick leave among HCWs in Kuwait and identified several demographic and occupational predictors of sick leave episodes and duration. Factors such as age, gender, nationality, managerial position, professions, and influenza vaccination were associated with variations in sick leave utilization. These findings provide population-level evidence relevant to workforce planning and occupational health strategies.

Evidence on sick leave patterns among HCWs in Kuwait remains limited. Previous reports have highlighted patterns of leave utilization and organizational influences on absenteeism within the public sector [15,16]. However, most prior studies were based on small samples or non-healthcare populations. The present nationwide analysis, therefore, contributes population-level evidence on demographic and occupational determinants of sickness absence among HCWs in Kuwait.

Slightly more than half of the HCWs experienced at least one sick leave episode during the study year. This prevalence is broadly comparable to findings reported from Saudi Arabia, although some hospital-based studies in the region have documented lower levels of sickness absence [3,17,18,19]. Differences across studies likely reflect variations in study design and sick leave certification practices.

Several regional studies, including those from Qatar and Lebanon [4,20], were limited to HCWs with documented sick leave, precluding estimation of the proportion with zero episodes. These findings underscore the value of population-based administrative data in capturing both sick leave users and non-users and supporting more accurate workforce planning.

The temporal distribution of sick leave episodes revealed distinct patterns between URTI-related and non-URTI-related absences, highlighting the influence of infectious disease dynamics on workforce absenteeism. URTI-related sick leave accounted for a substantial proportion of all sick leave episodes and demonstrated clear seasonal peaks during the winter months. This finding is consistent with evidence from studies conducted in Saudi Arabia, Lebanon, Qatar, Italy, and the United States [3,4,20,21,22], which have identified respiratory infections as a major contributor to sick leave among HCWs.

Moreover, the inverse association observed between influenza vaccination and sick leave in the present study is consistent with previous studies reporting lower absenteeism among vaccinated HCWs. This finding aligns closely with prior studies from Saudi Arabia, Italy, and the United States [3,21,22,23], which have similarly documented reductions in influenza-related absenteeism among vaccinated healthcare personnel. Despite its association with fewer sick leave episodes and days, seasonal influenza vaccination uptake among HCWs in Kuwait was unexpectedly low (7.31%). This level remains substantially below rates reported in neighboring countries, including Qatar (65.2%) and Saudi Arabia (50.8%) [24,25], as well as in developed settings such as the United States, where uptake exceeds 70% [26]. In contrast, influenza vaccination coverage among HCWs in Kuwait was considerably higher in earlier periods, reaching 67.2% in 2009 [27]. This marked decline observed in the post-COVID-19 period may reflect reduced prioritization of seasonal influenza vaccination following the pandemic.

In the present study, two occupational factors, profession and managerial position, were examined. Sick leave patterns varied significantly across professions, with medical technicians experiencing more frequent sick leave episodes and longer duration compared with nurses. These findings differ from prior studies in Saudi Arabia, Lebanon, Italy, and Iran, which reported higher sickness absence among nursing staff [3,4,19,21]. Such differences likely reflect differences in task structure, occupational exposures, workload intensity, and psychological demands across professions, as healthcare environments are often characterized by high job demands, emotional strain, and substantial professional responsibility, all of which may contribute to sickness absence among HCWs.

Managerial position was the second occupational factor assessed and was significantly associated with sick leave outcomes. This study is among the first population-level analyses in the region to quantify the impact of managerial positions, showing that HCWs in managerial positions experienced fewer sick leave episodes and shorter duration. This finding aligns with evidence from Italy, India, and Lebanon [4,8,21], where lower job grade, often representing non-managerial position, has been identified as a strong predictor of increased sickness absence. These findings underscore the importance of organizational hierarchy and working conditions, including differences in workload pressures, decision-making responsibility, and job control, beyond profession alone, in shaping sick leave patterns among HCWs. However, several workplace characteristics that may influence sickness absence (e.g., shift work schedules, workload intensity, facility type, and detailed job grade) were not available in the administrative dataset and, therefore, could not be incorporated into the present analysis.

Demographic characteristics showed patterns consistent with the previous literature. Female HCWs had higher sick leave utilization, a pattern widely reported across international studies [4,8,19,28]. Several explanations have been proposed for this consistent finding. Biological factors, including pregnancy-related health needs and gender differences, may affect susceptibility to certain infectious and musculoskeletal conditions. Social factors may also play a role, as female workers often carry a greater share of family caregiving responsibilities, which can influence patterns of work attendance and health-seeking behavior, particularly when combined with occupational stressors present in demanding healthcare environments. Younger HCWs demonstrated a higher sick leave burden, which may reflect greater involvement in frontline clinical roles and shift-based work schedules. Differences by nationality and place of residence may reflect variations in employment conditions, job security, or workforce distribution.

A major strength of this study lies in the use of nationwide administrative healthcare workforce records obtained from the MOH, which enhances the representativeness and generalizability of the findings across HCWs employed in Kuwait’s public healthcare sector. In addition, the use of ZINB regression models allowed for appropriate handling of over-dispersed sick leave count data with a high proportion of zero observations.

Nevertheless, several limitations should be acknowledged. First, although the analysis provides valuable population-level descriptive and analytical insights into sick leave patterns among HCWs in Kuwait, the temporal sequence between influenza vaccination and sick leave outcomes could not be fully established. Specifically, vaccination may not have preceded sick leave events; therefore, the observed relationship between vaccination status and sick leave outcomes should be interpreted as an association rather than a causal effect. In addition, diagnostic information was often recorded as free text rather than standardized codes, which may introduce some misclassification due to spelling variations or inconsistent terminology. Although data cleaning and a predefined keyword-based classification approach were used to identify URTI-related episodes, some misclassification may remain. Furthermore, several relevant occupational variables were not available in the administrative dataset and, therefore, could not be included in the regression models. These include work schedule characteristics (e.g., shift or night work), workload intensity, facility type (e.g., hospital vs. primary care), and detailed job grade. Because these workplace factors may influence sickness absence, their absence may have resulted in residual confounding.

The study findings have implications for workforce planning and occupational health policy in Kuwait. In particular, the low influenza vaccination uptake among HCWs in 2022, despite its observed association with fewer sick leave episodes and shorter duration, highlights the need to strengthen preventive occupational health strategies targeting HCWs. Governmental and healthcare authorities may consider implementing targeted workplace health programs, including enhanced vaccination campaigns, organizational support systems, and stress-reduction initiatives aimed at higher-risk workforce groups such as frontline staff and non-managerial personnel. Such measures may help mitigate sickness absence while improving workforce resilience and continuity of care.

Future research should examine the impact of recent policy changes, including the introduction of online self-certified sick leave and new attendance monitoring systems. Additional studies should also explore the role of occupational stress and burnout in shaping sickness absence among HCWs, particularly among frontline clinical staff and non-managerial personnel who demonstrated higher sick leave burden in this study. Incorporating validated measures of occupational stress, workload, and psychological strain would allow for a more precise understanding of the mechanisms linking workplace stressors and absenteeism.

## 5. Conclusions

This nationwide analysis identified several demographic and occupational predictors of sick leave among HCWs in Kuwait. Sick leave utilization was common and showed clear seasonal variation, with patterns associated with demographic and occupational characteristics and partly driven by URTI-related episodes. These findings provide the first population-level evidence on sick leave patterns and associated factors among HCWs in Kuwait. Moreover, the results underscore the value of administrative workforce data for monitoring sick leave and highlight the importance of strengthening influenza vaccination uptake and implementing targeted occupational health strategies for workforce groups with higher sick leave burden.

## Figures and Tables

**Table 1:** Characteristics of healthcare workers and sick leave distribution in Kuwait, 2022.

		Sick Leave Episodes ^†^	Sick Leave Duration (Days) ^†^
Variable	N (%)	Median [IQR]	Median [IQR]
Total	51,204 (100.00)	1 [5]	1 [8]
Age, mean (SD)	36.21 (10.06)		
Age Group			
≤29 years	13,983 (27.31)	1 [8]	1 [10]
30–39 years	20,596 (40.22)	1 [6]	2 [9]
40–49 years	11,347 (22.16)	1 [3]	1 [6]
≥50 years	5278 (10.31)	0 [1]	0 [3]
Gender			
Male	19,526 (38.13)	0 [2]	0 [3]
Female	31,678 (61.87)	2 [7]	3 [10]
Nationality			
Kuwaiti	15,021 (29.34)	2 [9]	3 [13]
Non-Kuwaiti	36,183 (70.66)	0 [4]	0 [6]
Place of Residence (Governorate)			
Farwaniya	15,436 (30.15)	1 [4]	1 [7]
Asimah	5942 (11.60)	1 [7]	2 [10]
Hawalli	13,743 (26.84)	0 [4]	0 [6]
Mubarak Al–Kabeer	2,234 (4.36)	2 [10]	3 [13]
Ahmadi	6969 (13.61)	1 [5]	1 [7]
Jahra	6880 (13.44)	1 [8]	1 [11]
Profession			
Nurses	20,511 (40.06)	1 [5]	2 [8]
Physicians	11,515 (22.49)	0 [1]	0 [2]
Dentists	2740 (5.35)	1 [5]	2 [7]
Pharmacists	2331 (4.55)	2 [7]	3 [10]
Medical Technicians	11,825 (23.09)	3 [11]	4 [14]
Other	2282 (4.46)	0 [3]	0 [6]
Managerial Position			
No	50,592 (98.80)	1 [5]	1 [8]
Yes	612 (1.20)	0 [3]	0 [6]
Influenza Vaccination			
No	47,461 (92.69)	1 [5]	1 [8]
Yes	3743 (7.31)	1 [4]	1 [6]

N: number of healthcare workers; SD: standard deviation; IQR: interquartile range; ^†^: a total of 196,840 sick leave episodes and 295,206 sick leave days were recorded in 2022. The total number of sick leave episodes (n = 196,840) includes six episodes that began in late 2021 and continued into 2022, which were counted in the annual episode totals.

**Table 2:** Predictors of sick leave episodes and sick leave duration among healthcare workers, Kuwait, 2022 (N = 51,204).

	Sick Leave Episodes			Sick Leave Duration (Days)		
Variable	IRR	95% CI	*p*-Value	IRR	95% CI	*p*-Value
Age (years)	0.968	0.968–0.970	<0.001 *	0.994	0.993–0.996	<0.001 *
Gender						
Male	Reference			Reference		
Female	1.22	1.18–1.26	<0.001 *	1.11	1.08–1.14	<0.001 *
Nationality						
Kuwaiti	Reference			Reference		
Non-Kuwaiti	0.52	0.50–0.54	<0.001 *	0.65	0.63–0.67	<0.001 *
Place of Residence (Governorate)						
Farwaniya	Reference			Reference		
Asimah	1.15	1.09–1.20	<0.001 *	1.08	1.04–1.14	<0.001 *
Hawalli	1.09	1.05–1.13	<0.001 *	0.96	0.93–1.00	0.048 *
Mubarak Al–Kabeer	1.25	1.17–1.33	<0.001 *	1.12	1.05–1.19	0.001 *
Ahmadi	1.01	0.97–1.06	0.568	0.93	0.89–0.97	<0.001 *
Jahra	1.56	1.50–1.63	<0.001 *	1.33	1.28–1.39	<0.001 *
Profession						
Nurses	Reference			Reference		
Physicians	0.37	0.35–0.39	<0.001 *	0.56	0.54–0.59	<0.001 *
Dentists	0.52	0.49–0.56	<0.001 *	0.60	0.57–0.64	<0.001 *
Pharmacists	0.78	0.73–0.83	<0.001 *	0.83	0.78–0.89	<0.001 *
Medical Technicians	1.11	1.07–1.15	<0.001 *	1.15	1.11–1.19	<0.001 *
Other	1.14	1.06–1.23	0.001 *	1.23	1.14–1.32	<0.001 *
Managerial Position						
No	Reference			Reference		
Yes	0.72	0.63–0.82	<0.001 *	0.79	0.70–0.90	<0.001 *
Influenza Vaccination						
No	Reference			Reference		
Yes	0.83	0.79–0.87	<0.001 *	0.85	0.81–0.89	<0.001 *

IRR: incidence rate ratio; CI: confidence interval; *: statistically significant at *p* < 0.05.

## Data Availability

The data used in this study are available from the corresponding author upon reasonable request but are not publicly available due to legal restrictions imposed by the MOH, Kuwait. Access to the data is subject to approval by the MOH.

## Supplementary Materials

The following supporting information can be downloaded at https://www.mdpi.com/article/10.3390/healthcare14060758/s1, Table S1: Model selections; Table S2: Inflation (logit) components of zero-inflated negative binomial regression models for sick leave episodes and sick leave duration among healthcare workers in Kuwait, 2022.

## Abbreviations

The following abbreviations are used in this manuscript:

HCW	Healthcare worker
MOH	Ministry of Health
IRR	Incidence rate ratio
CI	Confidence interval
URTI	Upper respiratory tract infection
SD	Standard deviation
IQR	Interquartile range
ZINB	Zero-inflated negative binomial
AIC	Akaike Information Criterion
BIC	Bayesian Information Criterion
KU	Kuwait University
HSCEC	Health Science Center Ethical Committee
