# Supplementary material for: Nationwide Patterns and Predictors of Sick Leave Among Healthcare Workers in Kuwait, 2022
Source: Healthcare (Basel). 2026 Mar 18;14(6):758. doi: 10.3390/healthcare14060758 (PMC13027212; doi:10.3390/healthcare14060758)
Supplement: Supplementary file 1 [file healthcare-14-00758-s001.zip › healthcare-4177891-supplementary.pdf]

Supplemental Table S1. Model selections

Outcome Variable	Model	Inflation variables	Log-likelihood	df	AIC	BIC
**Sick-leave episode**	Negative binomial	-	–109668.1	17	219370.3	219520.6
	**ZINB M1***	Age, Gender, Nationality, Place of Residence, Profession, Managerial Position, and Influenza Vaccination Status	–107533	33	215132	215423.9
	ZINB M2	Age, Gender, Nationality, Managerial Position, and Profession	–107585.7	27	215225.4	215464.2
	ZINB M3	Age Gender, Nationality, and Managerial Position	–108167.4	22	216378.9	216573.4
**Sick-leave duration**	Negative binomial	-	–124877.1	17	249788.2	249938.5
	**ZINB M1***	Age, Gender, Nationality, Place of Residence, Profession, Managerial Position, and Influenza Vaccination Status	–121318	33	242702	242993.9
	ZINB M2	Age, Gender, Nationality, Managerial Position, and Profession	–121352	27	242758	242996.8
	ZINB M3	Age Gender, Nationality, and Managerial Position	–122411.1	22	244866.2	245060.7

* Selected final model

Note: Lower AIC and BIC values indicate better model fits. The ZINB model, including all covariates in the inflation component (M1), demonstrated superior fit and was retained for the main analysis.

Supplemental Table S2. Inflate (logit) components of the zero-inflated negative binomial regression models for sick-leave episodes and sick-leave duration among healthcare workers in Kuwait, 2022, (N = 51,204).

	Sick-leave Episodes			Sick-leave Duration (days)		
**Variable**	**β**	**95% CI**	**P**	**β**	**95% CI**	**P**
**Age (years)**	–0.044	–0.049, –0.038	**<0.001***	–0.008	–0.010, –0.005	**<0.001***
**Gender**						
Male	Reference			Reference		
Female	–0.822	–0.881, –0.764	**<0.001***	–0.761	–0.808, –0.714	**<0.001***
**Nationality**						
Kuwaiti	Reference			Reference		
non-Kuwaiti	0.790	0.716, 0.863	**<0.001***	0.819	0.757, 0.882	**<0.001***
**Place of Residence (Governorate)**						
Farwaniya	Reference			Reference		
Asimah	–0.183	–0.288, –0.079	**0.001***	–0.109	–0.194, –0.025	**0.011***
Hawalli	–0.103	–0.183, –0.024	**0.011***	–0.110	–0.172, –0.048	**0.001***
Mubarak Al-Kabeer	–0.185	–0.325, –0.044	**0.010***	–0.193	–0.315, –0.071	**0.002***
Ahmadi	–0.116	–0.211, –0.022	**0.016***	–0.109	–0.183, –0.035	**0.004***
Jahra	–0.044	–0.131, –0.042	0.315	–0.062	–0.134, 0.011	0.094
**Profession**						
Nurses	Reference			Reference		
Physicians	1.475	1.375, 1.556	**<0.001***	1.515	1.445, 1.584	**<0.001***
Dentists	0.574	0.438, 0.711	**<0.001***	0.578	0.463, 0.693	**<0.001***
Pharmacists	0.572	0.441, 0.704	**<0.001***	0.481	0.367, 0.594	**<0.001***
Medical Technicians	0.392	0.313, 0.472	**<0.001***	0.353	0.290, 0.416	**<0.001***
Others	1.293	1.168, 1.418	**<0.001***	1.022	0.919, 1.126	**<0.001***
**Managerial Position**						
No	Reference			Reference		
Yes	1.07	0.77, 1.36	**<0.001***	0.864	0.660, 1.069	**<0.001***
**Influenza Vaccination**						
No	Reference			Reference		
Yes	–0.632	–0.784, –0.481	**<0.001***	–0.315	–0.408, –0.222	**<0.001***

*: Statistically significant at $p < 0.05$; CI, confidence interval; β: logit coefficients

Note: The table presents results from ZINB regression models and the logit coefficients (β) from the inflate (logit) components estimate the likelihood of belonging to the excess zero group. Positive coefficients indicate higher odds of belonging to the always-zero group, while negative coefficients indicate lower odds. All estimates are adjusted for variables listed.
